# Supplementary material for: Analysis of NS2-dependent effects on influenza PB1 segment extends replication requirements beyond the canonical promoter
Source: Nat Commun. 2025 Feb 22;16:1875. doi: 10.1038/s41467-025-57092-2 (PMC11846981; doi:10.1038/s41467-025-57092-2)
Supplement: Supplementary file 1 — Supplementary Information [file 41467_2025_57092_MOESM1_ESM.pdf]

Analysis of NS2-dependent effects on influenza PB1 segment extends replication requirements beyond the canonical promoter

Sharmada Swaminath[1,*], Marisa Mendes[1,*], Yipeng Zhang[1], Kaleigh A. Remick[2], Isabel Mejia[1], Melissa Güereca[1], Aartjan J.W. te Velthuis[2], Alistair B. Russell[1#]

[1]Department of Molecular Biology, School of Biological Sciences, University of California, San Diego, 9500 Gilman Drive, La Jolla, CA 92093, USA
[2]Lewis Thomas Laboratory, Department of Molecular Biology, Princeton University, Princeton, NJ 08544, USA
[*]These authors contributed equally to this work
[#]To whom correspondence should be addressed (a5russell@ucsd.edu)

Supplementary Figures and Tables

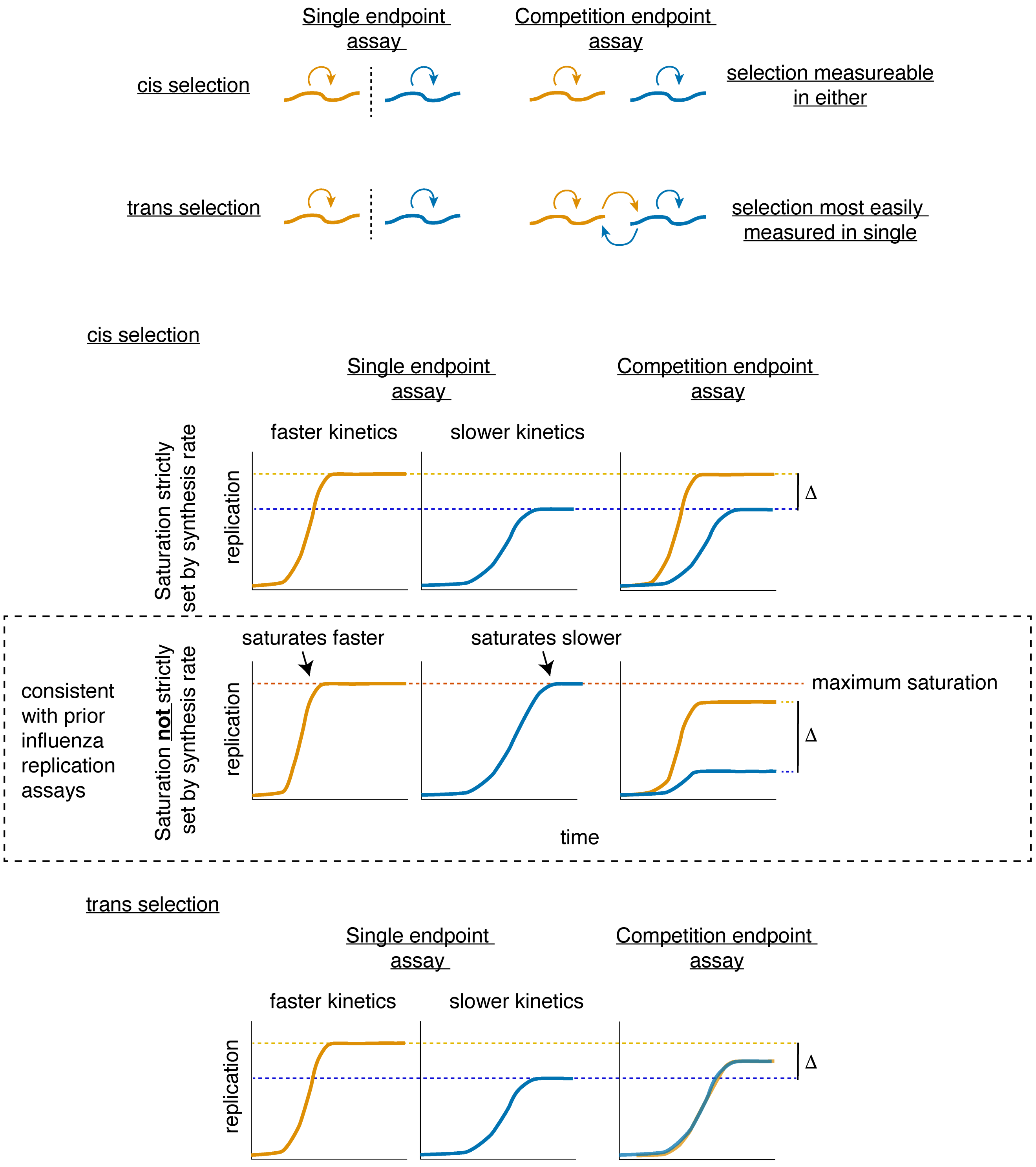

Supplementary Figure 1. Advantages and disadvantages of competition versus single endpoint assays in measuring viral replication.
Caption on following page.

Supplementary Figure 1. Advantages and disadvantages of competition versus single endpoint assays when measuring viral replication.
(Top) Single endpoint assays versus competition assays in conditions measuring *cis* versus *trans* selection pressures. If pressures act in *trans*, by combining variants in a single experiment, interactions become complex and difficult to relate back to individual variants. For variation that acts in *cis*, such as promoter sequences that impact replication initiation, both types of assays may relate diversity back to selection pressures. (Middle) For *cis* selection pressures, if steady-state, saturated, kinetics depend on synthesis rate alone, then either assay should appropriately capture changes to this rate. However, if they instead depend on some external factor (such as polymerase concentration), then it is possible that, when measured in single, those templates that replicate more slowly nevertheless achieve the same steady-state concentration. During a competition the final, steady state, concentration of each template should instead be sensitive to their initial rate of replication, allowing differences to be measured. Prior reports have demonstrated that influenza genomes compete for some rate-limiting substrate in minimal replication assays suggesting competition assays may give us greater sensitivity for the phenomena we specifically explore in this work.[47]

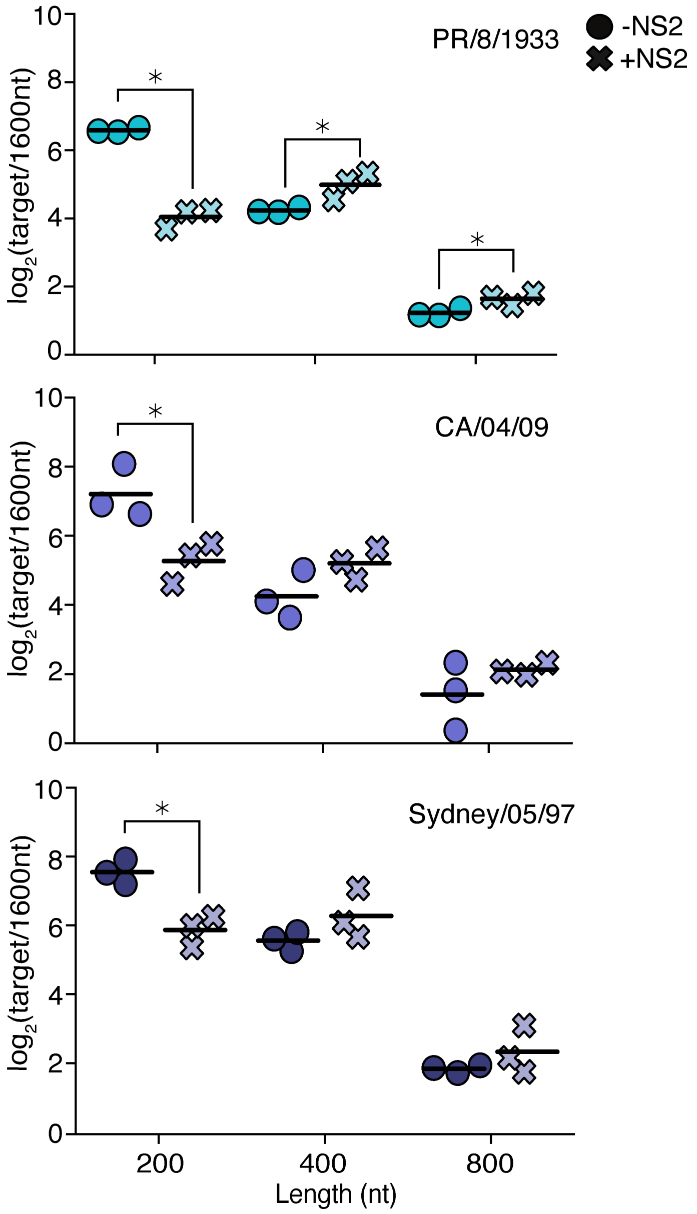

Supplementary Figure 2. NS2 expression from diverse IAV strains exhibit similar effects on genome replication.
Experiments performed as in Fig 2, using the PB1 segment and minimal replication machinery from A/WSN/1933 and NS2 from the indicated IAV strains. Asterisks indicate conditions significantly impacted by the expression of NS2, two-sample two-tailed t-test with a within-panel Benjamini-Hochberg corrected FDR <0.05. $n=3$, individual replicates and mean displayed. Source data are provided as a Source Data file.

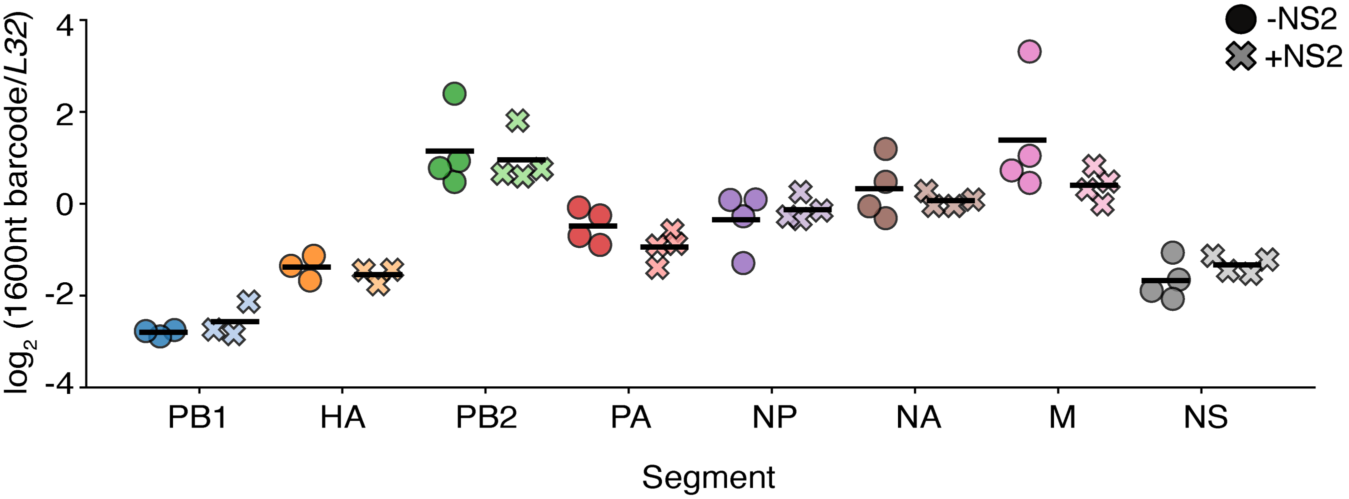

Supplementary Figure 3. Effects on 1600nt replication of NS2 expression on data from Fig 2. RNA from experiments presented in Fig 2 converted to cDNA using random hexamers and the housekeeping control, *L32* measured by qPCR. 1600nt values compared against *L32* to calculate whether final amounts of 1600nt variants were impacted by NS2 expression. No segment demonstrated significant enrichment or depletion upon NS2 expression (two-sample two-tailed t-test $p<0.05$). Source data are provided as a Source Data file.

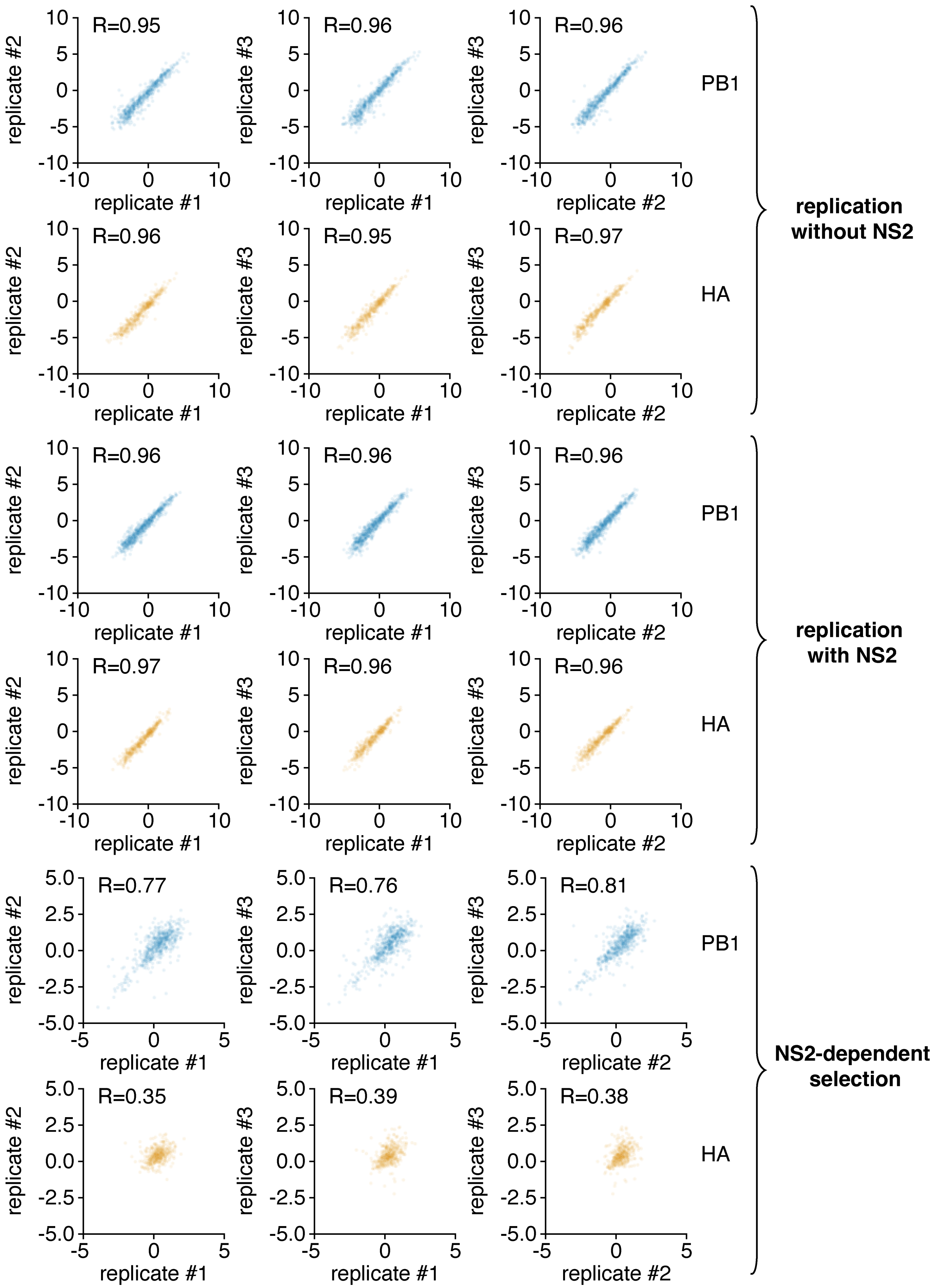

Supplementary Figure 4. Inter-replicate correlation from Fig. 3.
Inter-replicate enrichment or depletion values as calculated in Fig 3. Values are in $\log_2$. R is the Pearson correlation coefficient. Source data are provided as a Source Data file.

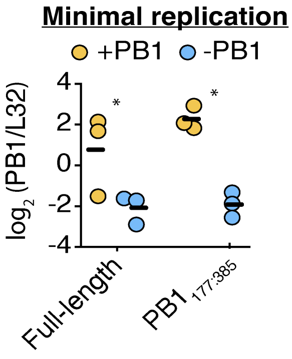

Supplementary Figure 5. PB1$_{177:385}$ exhibits consistent replication in minimal replication assays
Plasmids encoding indicated variants were transfected in minimal replication assays with, and without, PB1, and replication of the indicated template measured 24h post-transfection. Asterisks indicate significant replication in the presence of PB1 as compared to its absence, two-tailed t-test Benjamini-Hochberg corrected FDR <0.05. Source data are provided as a Source Data file.

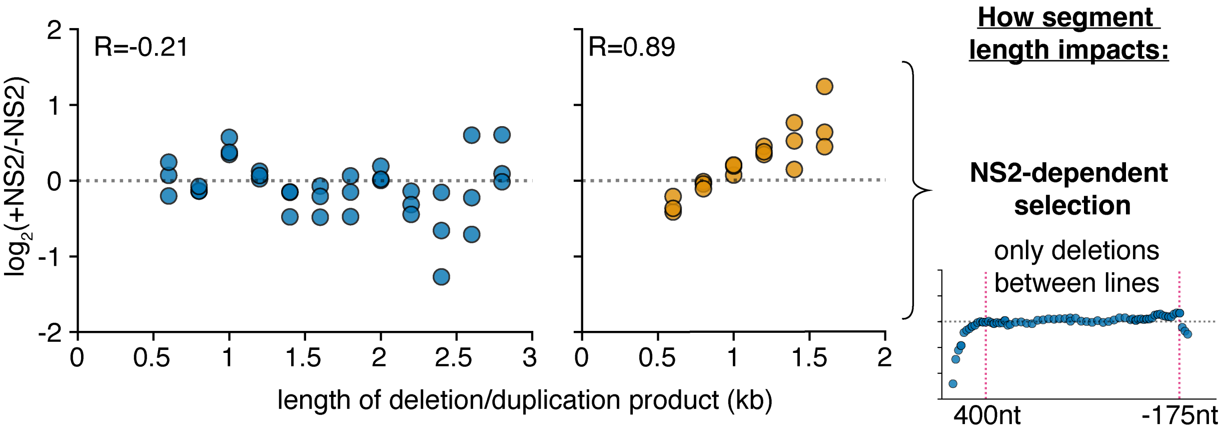

Supplementary Figure 6. Removal of NS2-responsive regions in PB1 does not produce data consistent with those observed in HA
Library data analysed as in Fig 3, bottom, excluding deletions that remove the indicated regions (first 400nt and last 175nt) in PB1 or HA. Source data are provided as a Source Data file.

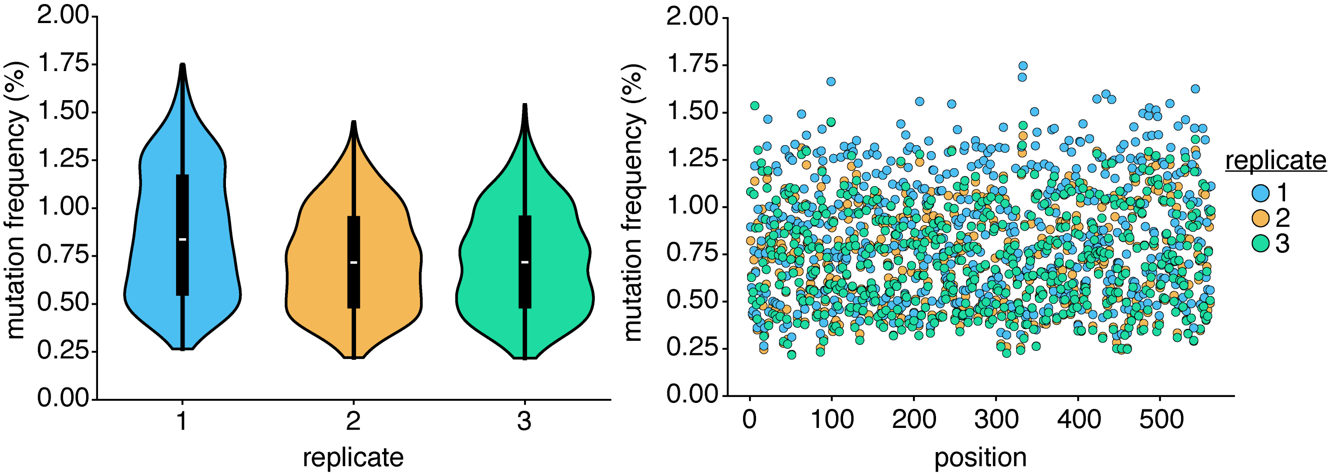

Supplementary Figure 7. Generation of single-nucleotide variant libraries in $PB1_{177:385}$.
(left) Per-site mutation frequency as measured by Illumina sequencing of $PB1_{177:385}$ libraries generated by mutagenic PCR across each of the three replicate libraries. Libraries were relatively uniform, with a median mutation frequency ranging from 0.716% to 0.836% (right) Data from (left) displayed per individual site in $PB1_{177:385}$. Mutation rate was relatively uniform across this template, with no particular regions exhibiting particularly aberrant frequencies across three replicates. Source data are provided as a Source Data file.

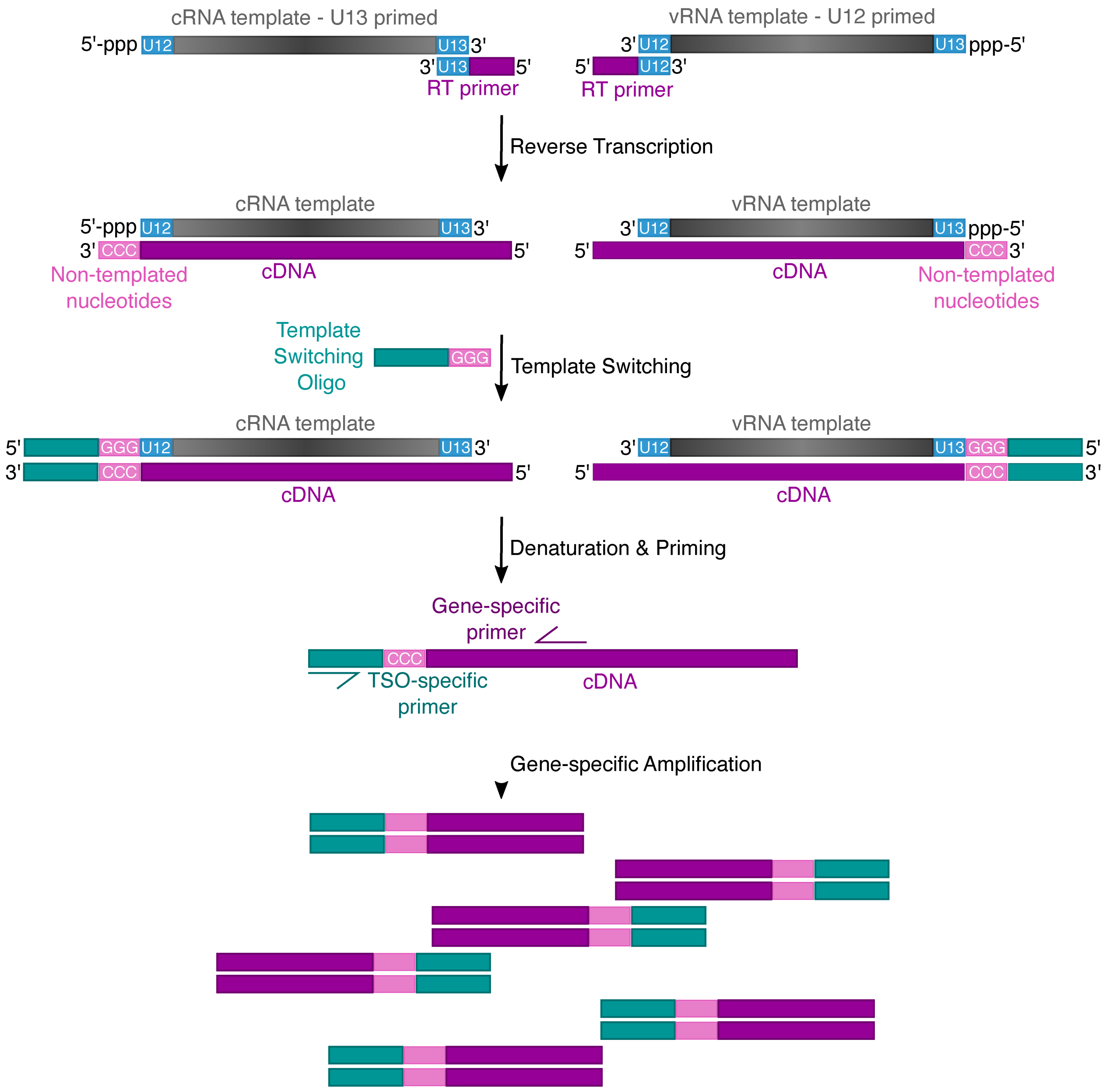

Supplementary Figure 8. RACE inference of 5' sequence.
Schematic depicting the process by which 5' sequence of cRNA and vRNA were measured. Source data are provided as a Source Data file.

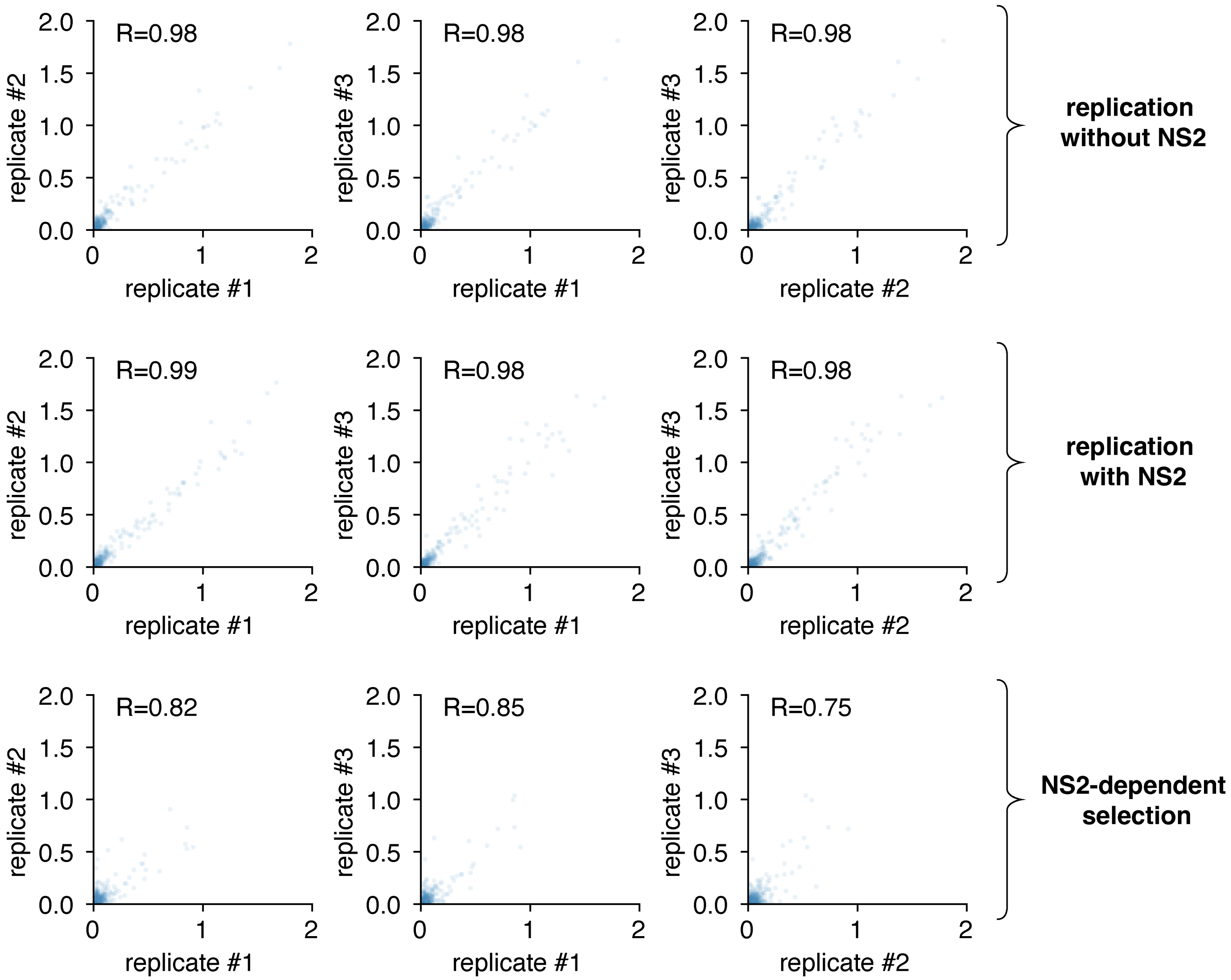

Supplementary Figure 9. Inter-replicate correlation of information content from Fig. 5.
Inter-replicate information content (total) values. Values are in bits. R is the Pearson correlation coefficient. Source data are provided as a Source Data file.

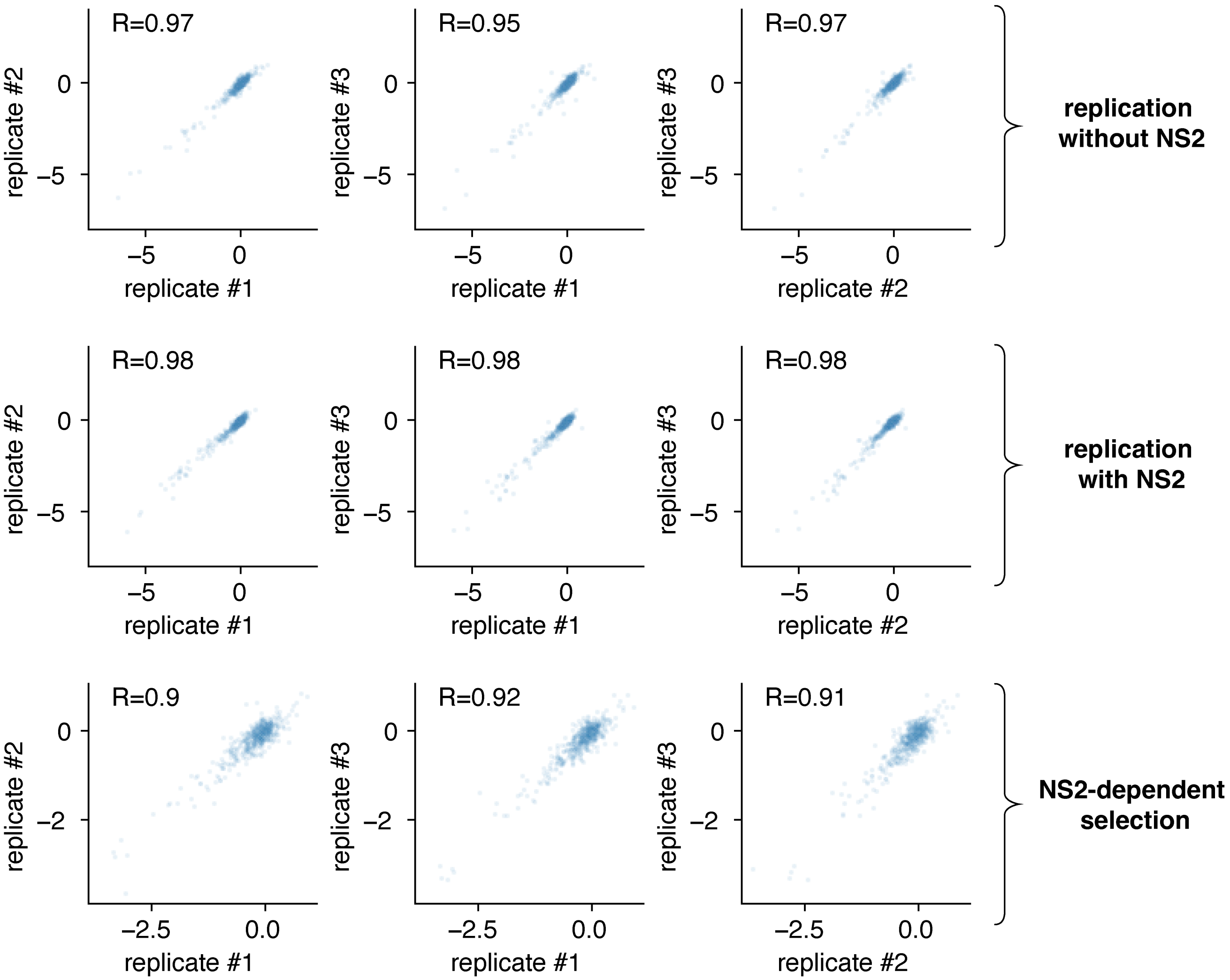

Supplementary Figure 10. Inter-replicate correlation from Fig. 5.
Inter-replicate selection on non-wild-type nucleotides as calculated in Fig 5. Values are in $\log_2$. R is the Pearson correlation coefficient. Source data are provided as a Source Data file.

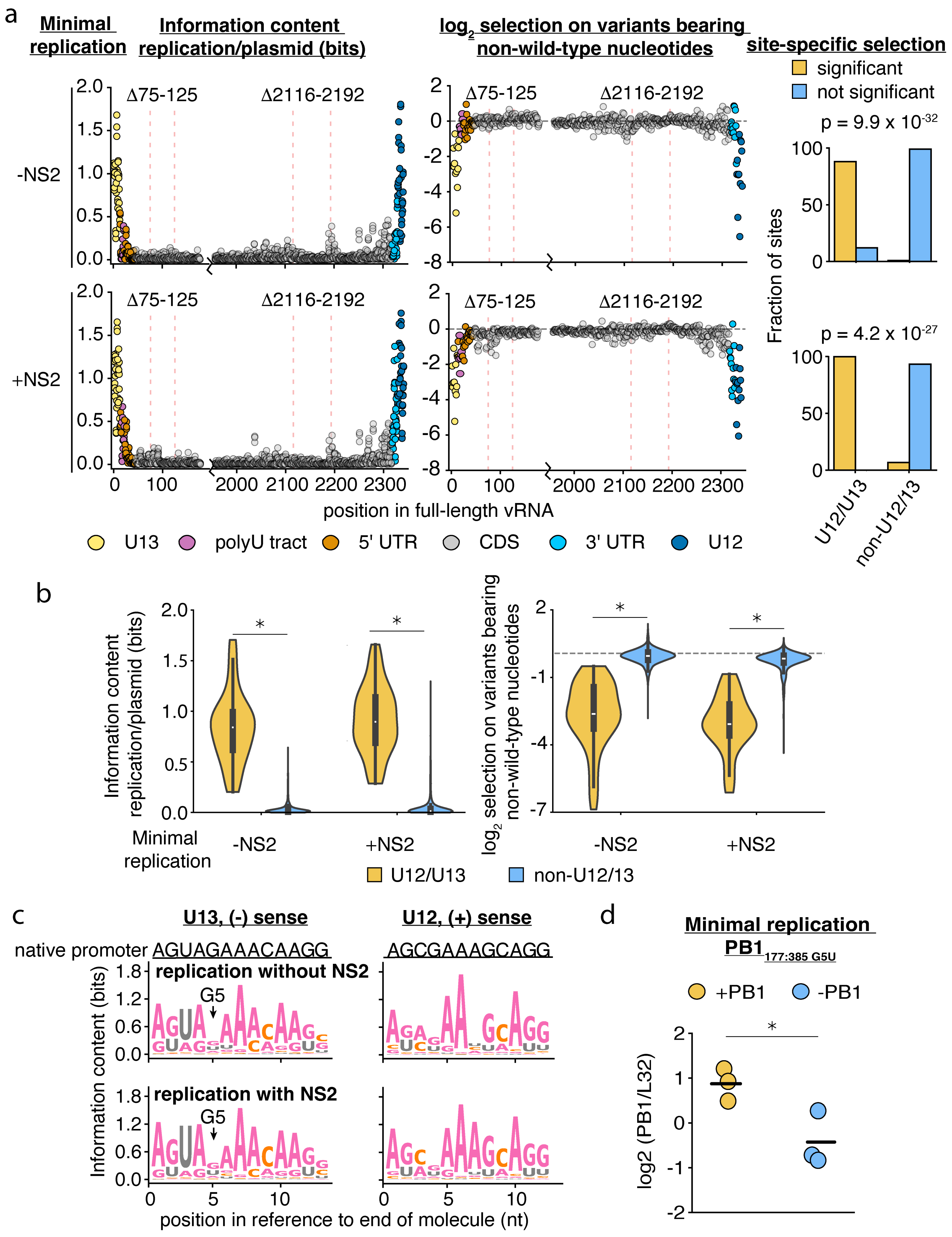

Supplementary Figure 11. Validation of the biological relevance of single-nucleotide variant libraries. Caption on following page.

Supplementary Figure 11. Validation of the biological relevance of single-nucleotide variant libraries **a**(left) Information content calculated using Shannon entropy of the selection preferences at each position under the indicated minimal replication conditions. (middle) Selection on non-wild-type nucleotides at each position under the indicated minimal replication conditions. Dotted line seperates positions where non-wild-type nucleotides exhibit higher replication (above) and those where they decrease replication. (right) Using selection on non-wild-type nucleotides, the fraction of sites under each category that met significance (at least two-fold effect size and exhibit non-zero selection one-sample two-tailed t-test with Benjamini-Hochberg corrected FDR of 0.1). Regions in the promoter are more likely to exhibit significance under all conditions, p values shown, Fisher's exact test. **b** (left) Distribution of information content values as shown in **a** (right) Distribution of selection on non-wild-type nucleotides as presented in **a**. For both panels, and under both conditions, promoter sequences were compared against non-promoter sequences by two-tailed two-sample t-test with Benjamini-Hochberg correction at an FDR of 0.05. **c** Sequence logos as generated from information content analysis as presented in **a**, only presenting the U12 and U13 sequences. The top nucleotide in each stack represents the highest preference. This nucleotide matches the wild-type sequence at all positions with the expression of NS2, and in all but three positions in the absence of NS2 expression. The indicated position is explored further. **d** Minimal replication assay with the indicated variant with, and without expression of PB1. Unlike other qPCR presented, this assay used a primer pair that cannot amplify the mRNA produced from our minimal replication expresion vector, to rule out that residual replication is due to mRNA from that PB1 mRNA expression vector. Significant replication was observed, two-sample one-tailed t-test, $p<0.05$, $n=3$. Source data are provided as a Source Data file.

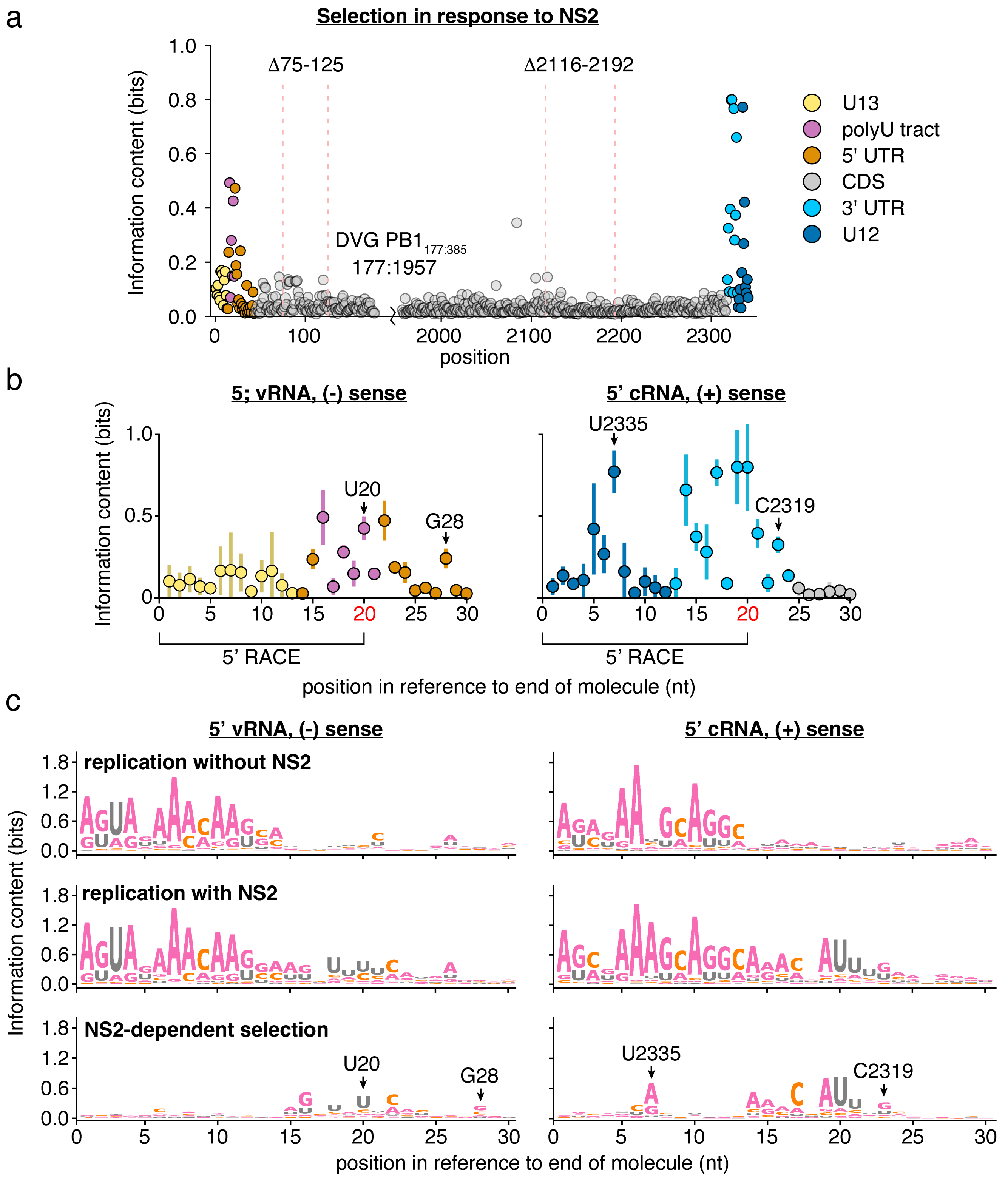

Supplementary Figure 12. Information content of single-nucleotide variant libraries in $PB1_{177:385}$. Information content calculated using Shannon entropy of the selection preferences at each position. Sequence logos generated by assigning this information content to each nucleotide in order of the strength of selection. **a** Information content analysis of data as presented in Fig. 5a. Each point represents the average of three replicates. **b** Information content analysis of data as presented in Fig. 5b, for NS2-dependent selection only. Mean and standard deviation displayed, n=3. Positions chosen for further analysis in Fig. 6 noted. **c** Sequence logo plots for positions displayed in Fig 5b. Data presented are the median values across three replicates. Positions chosen for further analysis in Fig. 6 noted. Inter-replicate correlation plots presented in (Supplementary Fig. 9) Source data are provided as a Source Data file.

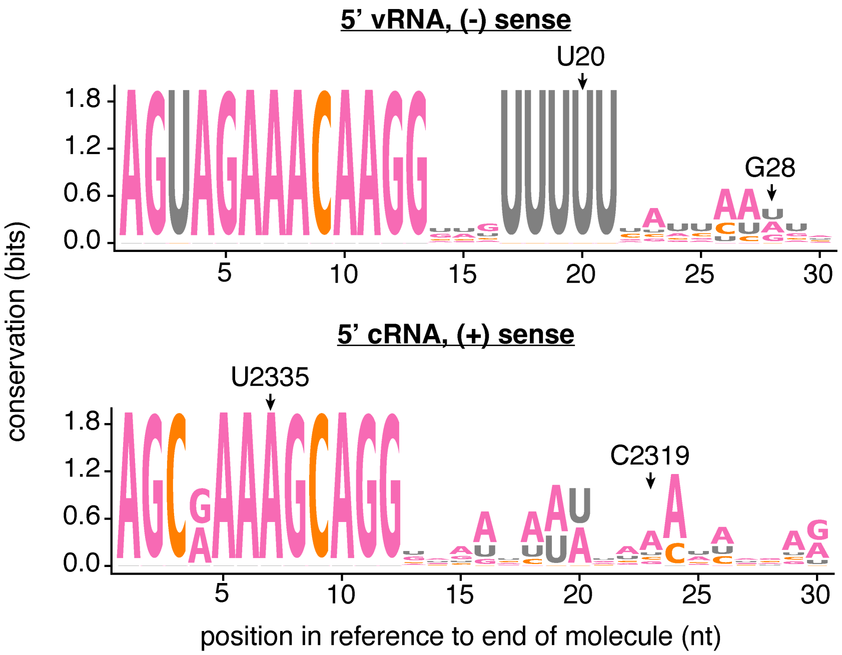

Supplementary Figure 13. NS2 responsive sites are not identical between IAV segments.
The first 30nt of the vRNA and cRNA were compared between the eight IAV segments, and sequence logo plots generated. The sites we tested in Fig. 6 are highlighted. Source data are provided as a Source Data file.

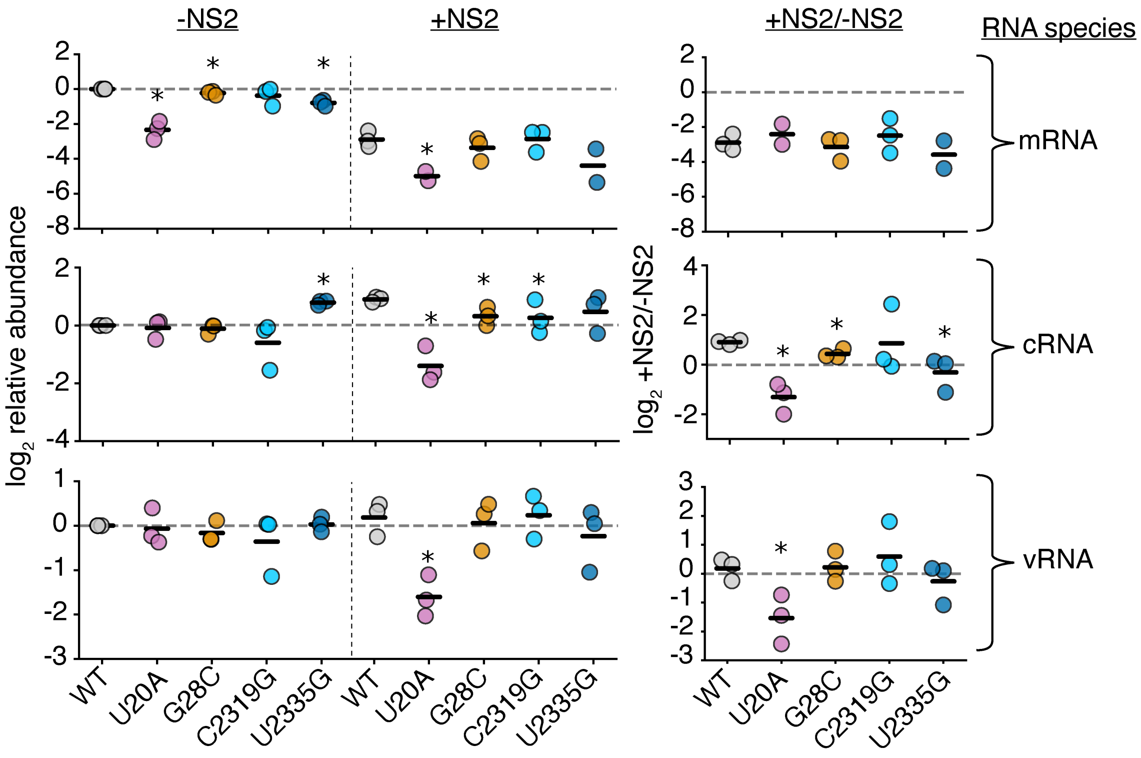

Supplementary Figure 14. Mutations to NS2-dependent sites can impact replication of genomes or antigenomes in full-length PB1

For all experiments, the canonical start codon was removed by mutagenesis to remove effects of additional expression of PB1. Quantitative analysis of primer extension performed as in Fig. 6, but with full-length template bearing mutations in the start codon rather than $PB1_{177:385}$. All values in left two columns corrected against a parental template in the absence of NS2. Dotted line represents that value, points above indicate an increase in that molecular species, below, decrease. Values in the right column represent the ratio of points between the left two columns. Asterisks indicate values that are significantly decreased relative to the parental template, one-tailed t-test with Benjamini-Hochberg corrected FDR <0.1. Individual replicates and mean presented, n=3. For several mRNA samples there was no signal detected above background, for these (U20A, U2335G) only detectable replicates presented. Source data are provided as a Source Data file.

context	Nucleotide position (vRNA)	log2 NS2-dependent effect	pval	qval
**5' UTR**	15	-1.26642623	0.0053062	0.05516478
**polyU**	16	-1.407887837	0.00580593	0.05530393
**polyU**	18	-1.795893957	0.00826029	0.06727946
**polyU**	20	-1.844711513	0.00254884	0.04325913
**polyU**	21	-1.37795038	0.00446839	0.05022535
**5' UTR**	22	-1.462633616	0.02373645	0.09737143
**5' UTR**	23	-1.285950966	0.01276786	0.07827812
**5' UTR**	28	-1.90659152	0.0048643	0.05257184
**CDS**	55	-1.101661611	0.00234029	0.04286222
**CDS**	56	-1.100544239	0.00189633	0.04232093
**CDS**	71	-1.326675414	0.01338237	0.07827812
**CDS**	72	-1.03733307	0.0000459	0.01290355
**CDS**	82	-1.178798373	0.0111679	0.07659062
**CDS**	83	-1.067901354	0.00338681	0.04531879
**CDS**	91	-1.372475945	0.00471253	0.05193021
**3' UTR**	2319	-1.555944694	0.00131708	0.03949276
**3' UTR**	2320	-1.015444989	0.02231737	0.09582604
**3' UTR**	2321	-1.696175204	0.0130107	0.07827812
**3' UTR**	2322	-3.011824531	0.0084403	0.06776352
**3' UTR**	2323	-3.149977188	0.00244055	0.04286222
**3' UTR**	2325	-3.02907342	0.00132183	0.03949276
**3' UTR**	2326	-1.928677286	0.02540826	0.09776512
**3' UTR**	2327	-1.56234586	0.00266523	0.04325913
**3' UTR**	2328	-3.048000255	0.00326677	0.04477866
**U12**	2335	-3.286629043	0.00325948	0.04477866
**U12**	2339	-1.072209116	0.0008218	0.03949276
**U12**	2340	-1.19881869	0.01855345	0.09035903

Supplementary Table 1. NS2-responsive sites.
Table of all sites identified as meeting significance from Fig 5a. Significance required a greater than two-fold effect size (positive or negative), p-values reported are from a one-sample t-test comparing against the null hypothesis of no selection. q-values represent Benjamini-Hochberg corrected FDR.
