# Supplementary material for: Analysis of NS2-dependent effects on influenza PB1 segment extends replication requirements beyond the canonical promoter
Source: Nat Commun. 2025 Feb 22;16:1875. doi: 10.1038/s41467-025-57092-2 (PMC11846981; doi:10.1038/s41467-025-57092-2)
Supplement: Supplementary file 2 — Description Of Additional Supplementary File [file 41467_2025_57092_MOESM2_ESM.pdf]

Description of additional supplementary files

Supplementary Data 1. Genome sequence of A/WSN/1933.
Genome sequence of A/WSN/1933 used in this study.

Supplementary Data 2. $PB1_{177:385}$ sequence.
Sequence of $PB1_{177:385}$ from this study.

Supplementary Data 3. NS2 sequences.
NS2 sequences used in this study.

Supplementary Data 4. Primer list.
List of all primer sequences used in this study.

Supplementary Data 5. PB1 sequences.
PB1 sequences used to assess natural diversity.
