# Supplementary material for: Analysis of NS2-dependent effects on influenza PB1 segment extends replication requirements beyond the canonical promoter
Source: Nat Commun. 2025 Feb 22;16:1875. doi: 10.1038/s41467-025-57092-2 (PMC11846981; doi:10.1038/s41467-025-57092-2)
Supplement: Supplementary file 8 — Reporting summary [file 41467_2025_57092_MOESM8_ESM.pdf]

nature portfolio

Corresponding author(s): Alistair Russell

Last updated by author(s): Feb 4, 2025

Reporting Summary

Nature Portfolio wishes to improve the reproducibility of the work that we publish. This form provides structure for consistency and transparency in reporting. For further information on Nature Portfolio policies, see our Editorial Policies and the Editorial Policy Checklist.

Statistics

For all statistical analyses, confirm that the following items are present in the figure legend, table legend, main text, or Methods section.

n/a	Confirmed	
☐	☒	The exact sample size (*n*) for each experimental group/condition, given as a discrete number and unit of measurement
☐	☒	A statement on whether measurements were taken from distinct samples or whether the same sample was measured repeatedly
☐	☒	The statistical test(s) used AND whether they are one- or two-sided<br>*Only common tests should be described solely by name; describe more complex techniques in the Methods section.*
☒	☐	A description of all covariates tested
☐	☒	A description of any assumptions or corrections, such as tests of normality and adjustment for multiple comparisons
☐	☒	A full description of the statistical parameters including central tendency (e.g. means) or other basic estimates (e.g. regression coefficient) AND variation (e.g. standard deviation) or associated estimates of uncertainty (e.g. confidence intervals)
☐	☒	For null hypothesis testing, the test statistic (e.g. *F*, *t*, *r*) with confidence intervals, effect sizes, degrees of freedom and *P* value noted<br>*Give P values as exact values whenever suitable.*
☒	☐	For Bayesian analysis, information on the choice of priors and Markov chain Monte Carlo settings
☒	☐	For hierarchical and complex designs, identification of the appropriate level for tests and full reporting of outcomes
☐	☒	Estimates of effect sizes (e.g. Cohen's *d*, Pearson's *r*), indicating how they were calculated

Our web collection on statistics for biologists contains articles on many of the points above.

Software and code

Policy information about availability of computer code

Data collection	No software used in collection.
Data analysis	The following software and package versions were used: Python 3.12.2, Trimmomatic 0.39, STAR 2.7.11b, Samtools 1.19, Samtools 1.19, FastQC 0.12.1, numpy 1.26.4, matplotlib 3.8.3, seaborn 0.13.2, pandas 2.2.1, scipy 1.12.0, and statsmodels 0.14.1.

For manuscripts utilizing custom algorithms or software that are central to the research but not yet described in published literature, software must be made available to editors and reviewers. We strongly encourage code deposition in a community repository (e.g. GitHub). See the Nature Portfolio guidelines for submitting code & software for further information.

Data

Policy information about availability of data

All manuscripts must include a data availability statement. This statement should provide the following information, where applicable:

- Accession codes, unique identifiers, or web links for publicly available datasets
- A description of any restrictions on data availability
- For clinical datasets or third party data, please ensure that the statement adheres to our policy

The processed files and raw sequencing data generated in this study have been deposited in the NCBI gene expression omnibus under accession GSE276697 (https://www.ncbi.nlm.nih.gov/geo/query/acc.cgi?acc=GSE276697), or additionally under bioproject PRJNA1158716 (https://www.ncbi.nlm.nih.gov/bioproject/PRJNA1158716). The

qPCR data concerning confirmation of individual variants, whether by transfection or viral infection, primer extension analysis data, and analysis of sequencing data as presented in this manuscript are all provided in the Source Data file.

Research involving human participants, their data, or biological material

Policy information about studies with human participants or human data. See also policy information about sex, gender (identity/presentation), and sexual orientation and race, ethnicity and racism.

Reporting on sex and gender	N/A
Reporting on race, ethnicity, or other socially relevant groupings	N/A
Population characteristics	N/A
Recruitment	N/A
Ethics oversight	N/A

Note that full information on the approval of the study protocol must also be provided in the manuscript.

Field-specific reporting

Please select the one below that is the best fit for your research. If you are not sure, read the appropriate sections before making your selection.

☒ Life sciences ☐ Behavioural & social sciences ☐ Ecological, evolutionary & environmental sciences

For a reference copy of the document with all sections, see nature.com/documents/nr-reporting-summary-flat.pdf

Life sciences study design

All studies must disclose on these points even when the disclosure is negative.

Sample size	Sample size provided throughout manuscript. We use the field-accepted n=3 as a minimum, at times using n=4 as resources allowed. While we performed no power calculations, within the variance we identify in each individual assay, we found this replicate structure to be sufficient. We additionally add that our mutagenesis libraries are a true n=3, independently generated during the mutagenesis steps and so avoiding unmeasured correlations from ideosyncracies in library preparation. This meets, and often exceeds, field standards.
Data exclusions	We chose not to test small effect sizes as described in each individual legend, as well as excluded data points where there was insufficient replicate support (also indicated explicitly in the legend for a given figure).
Replication	We orthogonally validated mutations of interest from our high-throughput screens across multiple, independent, experiments.
Randomization	Not relevant. Our high-throughput studies were "one-pot", meaning all variants were tested simultaneously and analyzed computationally such that rational bias by an experimenter cannot influence results. Our testing in single required labeled samples, but generally included internal controls that would have alerted us to any conscious or unconscious bias.
Blinding	Not relevant. As discussed under randomization, the "one-pot" and internally controlled nature of our experiments means this is fortunately not a concern.

Reporting for specific materials, systems and methods

We require information from authors about some types of materials, experimental systems and methods used in many studies. Here, indicate whether each material, system or method listed is relevant to your study. If you are not sure if a list item applies to your research, read the appropriate section before selecting a response.

Materials & experimental systems

n/a	Involved in the study
☒	☐ Antibodies
☐	☒ Eukaryotic cell lines
☒	☐ Palaeontology and archaeology
☒	☐ Animals and other organisms
☒	☐ Clinical data
☒	☐ Dual use research of concern
☒	☐ Plants

Methods

n/a	Involved in the study
☒	☐ ChIP-seq
☒	☐ Flow cytometry
☒	☐ MRI-based neuroimaging

Eukaryotic cell lines

Policy information about cell lines and Sex and Gender in Research

Cell line source(s)	AA549 cells from ATCC, 293T and MDCK-SIAT1 cells from Jesse Bloom.
Authentication	A549 cells have been authenticated by STR profiling. 293T and MDCK-SIAT1 cells have not been authenticated.
Mycoplasma contamination	All lines were routinely tested and found to be negative for mycoplasma by qPCR.
Commonly misidentified lines (See ICLAC register)	N/A

Plants

Seed stocks	*Report on the source of all seed stocks or other plant material used. If applicable, state the seed stock centre and catalogue number. If plant specimens were collected from the field, describe the collection location, date and sampling procedures.*
Novel plant genotypes	*Describe the methods by which all novel plant genotypes were produced. This includes those generated by transgenic approaches, gene editing, chemical/radiation-based mutagenesis and hybridization. For transgenic lines, describe the transformation method, the number of independent lines analyzed and the generation upon which experiments were performed. For gene-edited lines, describe the editor used, the endogenous sequence targeted for editing, the targeting guide RNA sequence (if applicable) and how the editor was applied.*
Authentication	*Describe any authentication procedures for each seed stock used or novel genotype generated. Describe any experiments used to assess the effect of a mutation and, where applicable, how potential secondary effects (e.g. second site T-DNA insertions, mosiacism, off-target gene editing) were examined.*
